# Intranasal Delivery of RIG-I Agonist Drives Pulmonary Myeloid Cell Activation in Mice

**DOI:** 10.3389/fimmu.2022.910192

**Published:** 2022-06-15

**Authors:** Sajith Nair, Yilun Wu, Trinh Mai Nguyen, Katja Fink, Dahai Luo, Christiane Ruedl

**Affiliations:** ^1^ School of Biological Sciences, Nanyang Technological University, Singapore, Singapore; ^2^ Lee Kong Chian School of Medicine, Nanyang Technological University, Singapore, Singapore

**Keywords:** RIG-I, innate immunity, intranasal vaccination, dendritic cells, monocytes, short hairpin RNA, influenza, type I interferon

## Abstract

Viral respiratory infections cause substantial health and economic burden. There is a pressing demand for efficacious vaccination strategies and, therefore, a need for a better understanding of the mechanisms of action of novel potential adjuvants. Here we investigated the effect of a synthetic RIG-I agonist, the dsRNA hairpin 3p10LA9, on the activation of pulmonary myeloid cells. Analysis of early innate immune responses revealed that a single intranasal 3p10LA9 dose induces a transient pulmonary interferon-stimulated gene (ISG) and pro-inflammatory cytokine/chemokine response, which leads to the maturation of three distinct dendritic cell subpopulations in the lungs. While lung resident dendritic cell decrease shortly after 3p10LA9 delivery, their numbers increase in the draining mediastinal lymph node, where they have migrated, maintaining their activated phenotype. At the same time, dsRNA hairpin-induced chemokines attract transiently infiltrating monocytes into the lungs, which causes a short temporary pulmonary inflammation. However, these monocytes are dispensable in controlling influenza infection since in CCR2 deficient mice, lacking these infiltrating cells, the virus load was similar to the wild type mice when infected with the influenza virus at a sublethal dose. In summary, our data suggest that intranasal delivery of dsRNA hairpins, used as a RIG-I targeting adjuvant, represents an attractive strategy to boost type I inteferon-mediated lung dendritic cell maturation, which supports viral reduction in the lungs during infection.

## Introduction

The current threat of SARS-CoV-2 pandemic to humanity and possible newly emerging respiratory viruses in the future underline the urgency for the development of preventive strategies against acute viral respiratory infections. Thus, there is an urgent demand for novel, efficient vaccination strategies, including the design of effective vaccine adjuvants capable of boosting innate as well as adaptive host immunity. Also, establishing alternative routes of vaccine administration beyond the “classic” systemic delivery is needed to boost local immunity and to offer easier and possibly more accepted alternative options. In the context of respiratory viruses, mucosal immunity in the upper respiratory tract and lung can help block infection at the point of infection (1–3).

Both innate and adaptive immune responses are essential for the development of efficient vaccine strategies. Dendritic cells (DCs) sense danger signals *via* the pathogen’s pattern recognition receptors (PRRs) including the toll-like receptor (TLR) and the retinoic acid-inducible gene I (RIG-I) like receptor (RLR) families (4) supporting the activation and expansion of pathogen-specific T- and B-cells (5). RIG-I recognizes evolutionarily conserved Pathogen-Associated Molecular Patterns (PAMPs) associated with invading pathogens, specifically short hairpin double-stranded RNA (dsRNA) with a 5’-PPP terminus. Its ligation initiates strong innate immune responses *via* the induction of type I interferon (IFN) expression (6–10). Type I IFNs induce multiple anti-viral programs in cells expressing the type-I interferon receptor. In particular, the expression of genes known as the Interferon Stimulated Genes (ISGs) generate an anti-viral cell state that efficiently curtails viral spread in infected tissue and initiates innate and adaptive immune responses (11, 12). Consistent with its role as a PRR, RIG-I is expressed in various cell types at the host-environment interface, not just limited to immune cells but is also expressed in cells of epithelial, endothelial and mesenchymal origin, positioned ideally to detect invading pathogens and to activate downstream antiviral responses (13). In mouse lungs, epithelial cells, endothelial cells and inflammatory macrophages are shown to have the highest expression levels of RIG-I (14). Thus, RIG-I is a critical component of the host innate immune system, which acts as the first line of defence against pathogens and aids in the rapid and efficient detection of several viruses across different families, including Orthomyxovirus, Flavivirus, Coronavirus, Herpesvirus, and Hepadnavirus (13).

Several targets for adjuvants have been proposed, including TLRs, c-type lectins, and cytosolic PRRs (14). Recently, targeting cytosolic-nucleic acid-sensing pathways with specific RIG-I ligands emerged as promising therapeutics for cancer treatment (15). The RIG-I-mediated boosting of the adaptive response can be imitated with artificial RIG-I binding molecules and can be leveraged to induce efficient vaccine responses by using such molecules as adjuvants. Several types of RNA molecules of different lengths have been tested in mice and have shown the protective capacity in the context of influenza, SARS-CoV-2, and vesicular stomatitis virus model vaccine (16–18). RIG-I targeting molecules have shown efficacy in mouse models (15, 19). Ongoing clinical trials test RIG-I ligands as adjuvants for a rabies vaccine and in cancer treatments in combination with checkpoint blockade antibodies providing first information on the safety profile and potential efficacy of such molecules in humans (20).

We and others have previously described short dsRNA hairpins (called immune-modulatory RNA molecules - immRNA) that bind specifically to RIG-I and induce anti-viral responses (21, 22). These immRNAs are 5’ phosphorylated short RNA with 10 paired bases and a panhandle that efficiently activate the induction of type I IFNs in cell lines, in mice, and in primary human cells (22, 23). In the context of Influenza A Virus (IAV) infection, earlier studies on mice have shown that RIG-I agonists are able to induce a potent humoral response, provide protection against IAV infection and induce the expression of a wide range of type-I IFN-mediated anti-viral genes including Interferon Stimulated Genes (ISG) and inflammatory cytokines (1, 18, 24–27). Since pulmonary myeloid cells are one of the first immune cells to come in contact with IAV at the site of infection and are known to act as the bridge between innate and adaptive immune responses it is essential to study in detail how these cells respond *in vivo* to RIG-I agonists.

Here, we investigated the impact of intranasal dsRNA delivery specifically on myeloid lung cells. We performed a detailed flow cytometry analysis of the myeloid cellular landscape in the lower respiratory tract at different time points post-inoculation using a synthetic RIG-I agonist, the dsRNA hairpin 3p10LA9. We have documented previously that alveolar macrophages (AM), although essential to block the virus’ spread, do not affect the adaptive response (28). In contrast, CD103^+^ DCs are required to induce protective CD8^+^ T cells as part of the viral-specific adaptive immune response (29). Due to the importance of DCs in regulating anti-viral immune responses, we have focused on the effect of dsRNA hairpins on distinct pulmonary DC subpopulations. We found that intranasal delivery of 3p10LA9 induced a strong transient ISG response in the lower respiratory tract and supported lung DC maturation. Shortly after 3p10LA9 intranasal treatment, the numbers of matured DCs in lungs decrease while their numbers increase in the lung draining LNs, where they show an activated phenotype. Furthermore, intranasal delivery of 3p10LA9 significantly reduced viral load upon challenge with IAV. Together, our results demonstrate that dsRNA hairpins are promising intranasal vaccine adjuvants against respiratory viral infections.

## Materials and Methods

### Ethics Statement

All studies involving mice were carried out in strict accordance with the recommendations of the National Advisory Committee for Laboratory Animal Research and all protocols were approved by the Institutional Animal Care and Use Committee of the Nanyang Technological University (ARF-SBS/NIE A19050 and A-0375).

### Mouse Strains

C57BL/6J and CCR2^-/-^ (B6.129S4-*Ccr2* ^tm1lfc^/J) mice were obtained from The Jackson Laboratory (USA). Mice were bred and maintained in the specific pathogen-free animal facility of the Nanyang Technological University (Singapore). Only female 6- to 8-week-old mice with at least 19 g body weight were used for the *in vivo* experiments.

### Cell Line

A549-Dual™ cells were purchased from *In vivo*Gen, USA and cultured in Dulbecco’s Modified Eagle Media (DMEM, Thermo Fisher Scientific, USA) supplemented with 10% fetal bovine serum (FBS, Biosera, France), penicillin-streptomycin (Thermo Fisher Scientific) in a humidified incubator at 37°C with 5% CO_2_.

### Synthesis of RIG-I Agonists

The immRNA 3p10LA9 (A9) was produced using T7 RNA polymerase, followed by purification with Hi-Trap Q HP column (Cytiva, MA, USA) (23). Briefly, the A9 DNA template (IDT, Singapore) was annealed 95°C for 5 min and slowly cooled to room temperature. Subsequently, the transcription was performed by adding 1 µM A9 template into reaction buffer (40 mM HEPES, pH7.5, 30 mM MgCl_2_, 2 mM spermidine, 10 mM DTT, 5 mM GTP, 4 mM CTP/ATP/UTP, and 0.01% Triton-X100, pH 7.4) containing 400-600 nM T7 polymerase and 0.2 U/ml thermostable inorganic pyrophosphatase (New England BioLabs, MA, USA) for 24h at 37°C. RNA products were extracted using phenol/chloroform/isoamyl alcohol (v/v/v = 25/24/1, Sigma, MO, USA) and precipitated in 75% (v) ethanol and 0.1% (v/v) sodium acetate (0.3 M, pH 5.2). All precipitated RNAs were dissolved in HEPES buffer (10 mM, pH 7.4) and isolated by Hi-Trap Q-Hp column for purification. The obtained A9 RNA was precipitated, re-dissolved in RNase-free H_2_O, and stored at -80°C for further use. All other RIG-I agonists (3p10L, 3p10LG9, SLR14, SLR14A9) were synthesized using the same method with different DNA templates. The sequence of all RIG-I agonists, along with their references are shown in **Table 1** and the sequence of the DNA template used for their *In Vitro* Transcription (IVT) in **Table 2**.

**Table 1:** List of RNAs.

Name	Sequences (5’-3’)	References
3p10L	ppp GGACGUACGU UUCG ACGUACGUCC	Kohlway et al., 2013
3p10LA9	ppp GGAUUUCCACCUUCGGGGGAAAUCC	Yong et al., 2019 (23)
3p10LG9	ppp GGAUUUCCGCCUUCGGGGGAAAUCC	Yong et al., 2019 (23)
SLR14	ppp GGAUCGAUCGAUCGUUCGCGAUCGAUCGAUCC	Linehan et al., 2018 (30)
SLR14A9	ppp GGAUCGAUACGAUACGUUCGCGAUCGAUCGAUCC	In this paper

**Table 2:** List of DNA Templates for IVT.

Name	Sequences (5’-3’)
3p10L	RGTAATACGACTCACTATAGGATTTCCCCTTCGGGGGAAATCC FGGATTTCCCCCGAAGGGGAAATCCTATAGTGAGTCGTATTAC
3p10LA9	RGTAATACGACTCACTATAGGATTTCCACCTTCGGGGGAAATCC FGGATTTCCCCCGAAGGTGGAAATCCTATAGTGAGTCGTATTAC
3p10LG9	RGTA ATA CGA CTC ACT ATA GGA TTT CCG CCT TCG GGG GAA ATC CF GGA TTT CCC CCG AAG GCG GAA ATC CTA TAG TGA GTC GTA TTA C
SLR14	RGTAATACGACTCACTATA GGATCGATCGATCGTTCGCGATCGA TCGATCCFGGATCGATCGATCGCGAACGATCGATCGATCCTATAGTGAGTCGTATTAC
SLR14A9	R GTAATACGACTCACTATAGGATCGATaCGATCGTTCGCGATCGA TCGATCCFGGATCGATCGATCGCGAACGATCGATCGATCCTATAGTGAGTCGTATTAC

### Type I IFN Reporter Assay

A549-Dual™ cells (50,000 cells/well) were seeded in a 96-well plate. Each dsRNA hairpin (2 µM) was incubated with equal volume of LyoVec™ (*In vivo*Gen, USA) for 20 min at room temperature following supplier’s instructions. The complex RNA-LyoVec was diluted and transfected onto the cells at a final concentration of 100 nM and 10 nM, respectively. The luciferase in the supernatant was detected by the QUANTI-Luc reagent (*In vivo*Gen, San Diego, CA, USA). Luminescence was measured on a Synergy H1 microplate reader (Biotek, Winooski, VT, USA) after 24h incubation.

### Generation of Bone Marrow-Derived Dendritic Cells (BMDCs)

Bone marrow (BM) cells were flushed out from femur and tibia. After red blood cell lysis using 0.89% Ammonium Chloride, 30 million BM cells were seeded in a 10 cm^2^ tissue culture dish in 15 ml of IMDM and 2%FBS containing 200 ng/ml Flt3L, with media topped up every third day. Non-adherent cells were harvested on day 9 to obtain a culture enriched for Siglec H^+^ pDCs and CD11b^+^ cDC2. To obtain a culture enriched for CD103^+^ DCs and CD11b^+^ DCs, 20 million BM cells were seeded in a 10 cm^2^ tissue culture dish in 20 ml of IMDM +2% FBS in the presence of 200 ng/ml Flt3L and 5 ng/ml GM-CSF, with media topped-up every third day, non-adherent cells were re-seeded into a new culture dish on the sixth day and non-adherent cells were finally harvested on day 12.

### Transfection of BMDCs With Distinct dsRNA Hairpins

BM-derived DC subpopulations (1x10^6^/well) were seeded in a 24-well plate. Lipofectamime™ 2000/dsRNA hairpins complexes were prepared according to the manufacturer’s instructions (Thermo Fisher Scientific, Waltham, MA, USA) and tested on all three DC subpopulations at different concentrations (0.1-100 nM). Poly (I:C) (5 μg/ml) was used as a positive control. Twenty-four hours later, cells were harvested and stained with anti-CD86 antibody and subsequently analysed by flow cytometry.

### Intranasal Delivery of RIG-I Agonist

Twenty-five µg of 3p10LA9 was complexed with the transfection reagent *in vivo*-JetPEI^®^ (Polyplus-transfection, Illkirch, France) following the manufacturer’s instructions. Twenty-five µl were delivered intranasally to each mouse anesthetized prior with Ketamine (10 mg/kg body weight) and Xylazine (2 mg/kg body weight). Control mice were injected with *in vivo*-JetPEI^®^ vehicle alone.

### Influenza Virus Infection

Influenza virus strain PR8 (A/Puerto Rico/8/1934 (H1N1) was used in all virus infection experiments and was a gift from Prof Mike Kemeny. Each mouse was anesthetized using Ketamine (10 mg/kg body weight) and Xylazine (2 mg/kg body weight) prior to intranasal delivery of 10^2^ plaque-forming units (pfu) of the virus suspended in 20 µl PBS. Infection was assessed by quantifying the virus titers by measuring the relative RNA levels of the M1 viral protein in IAV infected mouse lung tissues compared to uninfected lung tissues by real-time qPCR on day 6 post-infection (29).

### Isolation of Lung and Mediastinal LN Cells

Euthanized mice were perfused intracardially with 10 ml cold PBS. The lungs and mediastinal lymph node (mLN) were dissected, followed by 1 h incubation in IMDM containing collagenase D (1 mg/ml, Roche, Basel, Switzerland) and DNase I (50 µg/ml, Roche) at 37°C. Then a single cell suspension was obtained by meshing the digested tissues through a cell strainer. To further enrich the leukocytes, lung cell suspensions were resuspended in 5 ml of 35% Percoll™ (GE Healthcare Life Science, Chicago, IL, USA) before centrifuging at 600 ×*g* for 10 min at room temperature (RT). Red blood cell lysis was performed using 0.89% ammonium chloride solution.

### Lung and LN DC Analysis by Multi-Parameter Flow Cytometry

The single-cell suspensions from lung/mLN were incubated with anti-Fc receptor antibody (2.4G2) and DAPI (1:1000 dilution) for 20 min at 4°C. After washing, the cells were stained with antibody cocktails for 25 min at 4°C. For DC profiling the following antibodies were used: Buv395-labelled anti B220 (clone RA3-6B2), Buv737-labelled anti CD45 (clone 30-F11), Buv421-labelled anti CD169 (clone 3D6.112), BV510-labelled anti-MHC II (clone M5/114.15.2), BV570-labelled anti Ly6C (clone HK1.4), BV785-labelled anti CD11c (clone N418), FITC-labelled anti Siglec-H (clone 551), PerCP-eF700-labelled anti CD103 (clone 2E7), PE-cy7-labelled anti F4/80 (clone BM8), APC-labelled anti CD64 (clone X54-4/7.1), PE-labelled CD86 (clone GL1 and APC-Cy7-labelled anti CD11b (clone M1/70). All antibodies, except for anti-MHC II (1:000) and anti-Ly6C (1:800), were diluted 1:600 for staining if not mentioned otherwise. All antibodies were purchased from Biolegend (San Diego, CA, USA).

### IFN-α ELISA

Bronchoalveolar Lavage **(**BAL) and serum were collected from mice 14 h after intranasal treatment with vehicle alone or 3p10LA9. IFN-α ELISA was conducted according to the manufacturer’s instructions (Invitrogen, Waltham, WA, USA).

### Purification of BM-Derived and Lung Myeloid Cell Subpopulations

To obtain purified DC/macrophage subpopulations, BM-derived DCs and isolated lung cells were stained with anti-MHC II, anti-CD11c, anti-CD11b, anti-CD103, anti-F4/80 and anti-Siglec H and sorted using the BD FACSAria cell sorter.

### RNA Extraction, cDNA Synthesis, and Real-Time PCR Analysis

Mouse lung tissue was harvested into TRIzol reagent (Thermo Fisher Scientific, Waltham, MA, USA) and homogenized immediately. Total cellular RNA was then isolated using the RNAsimple Total RNA Kit (TIANGEN Biotech, Beijing, China) following the manufacturer’s instructions. The quality of RNA was assessed spectrophotometrically by ascertaining that the ratios of A260/280 and A260/230 were higher than 2.

Two µg RNA was used as template to synthesize cDNA using M-MLV Reverse Transcriptase (PROMEGA, Madison, WI, USA) following the manufacturer’s instructions. Real-time PCR was performed using the Precision FAST qPCR Master Mix (Primerdesign Ltd., Cambridge, UK).

The primer sequences used for the genes are:

*Beta-Actin* Fwd: AAGGCCAACCGTGAAAAGAT, *Beta-Actin* Rev: CCTGTGGTACGACCAGAGGCATACA; Virus *M1* Fwd: GGACTGCAGCGTTAGACGCTT, Virus *M1* Rev: CATCCTGTTGTATATGAGGCCCAT; *Rig-I* Fwd: CCACCTACATCCTCAGCTACATGA, *Rig-I* Rev: TGGGCCCTTGTTGTTCTTCT; *Isg15* Fwd: TCTGACTGTGAGAGCAAGCAG, *Isg15* Rev: ACCTTTAGGTCCCAGGCCATT; *Iftim3* Fwd: GCCTACTCCGTGAAGTCTAGG, *Iftim3* Rev: AATGGTGATAACAACCATCAGGA; *Rsad2* Fwd: CCCCCGTGAGTGTCAACTAC, *Rsad2* Rev: GATCTTCTCCAAACCAGCCTGT; *Bst2* Fwd: CCTGTAGAGACGGGTTGCG, *Bst2* Rev: CTGAAGGGTCACCACGGTC; *Ccl2* Fwd: CACTCACCTGCTGCTACTCA, *Ccl2* Rev: GCTTGGTGACAAAAACTACAGC; *Ccl5* Fwd: GTGCCCACGTCAAGGAGTAT, *Ccl5* Rev: TCGAGTGACAAACACGACTG; *Ccl7* Fwd: CCCTGGGAAGCTGTTATCTTCAA, *Ccl7* Rev: CTCGACCCACTTCTGATGGG; *Il6* Fwd: CTCTGGGAAATCGTGGAAAT, *Il6* Rev: CCAGTTTGGTAGCATCCATC; *Tnfα* Fwd: TGGAGCAACATGTGGAACTC, *Tnfα* Rev: GTCAGCAGCCGGTTACCA; *Il1b* Fwd: GGGCCTCAAAGGAAAGAATC, *Il1b* Rev: TTCTTCTTTGGGTATTGCTTGG; *Cxcl9* Fwd: CTCGGACTTCACTCCAACACA, *Cxcl9* Rev: ATCACTAGGGTTCCTCGAACT; *Cxcl10* Fwd: CCACGTGTTGAGATCATTGCC, *Cxcl10* Rev: TCACTCCAGTTAAGGAGCCC.

### Statistical Analysis

Statistical analysis was performed using GraphPad Prism 9.0.1 software (GraphPad Software, La Jolla, CA, USA). All values were expressed as the mean ± standard error of the means (SEM). Samples were analyzed by Student’s *t*-test (two-tailed) and one-way ANOVA with Tukey’s multiple comparison test. A *P*-value <0.05 was considered to indicate statistical significance. The number of animals used per group is indicated in the figure legends as ‘‘n.’’ The replications for each experiment is mentioned in the legend.

## Results

### dsRNA Hairpins Induce Type I Interferon Response in a Reporter Cell Line

**Figure 1:**
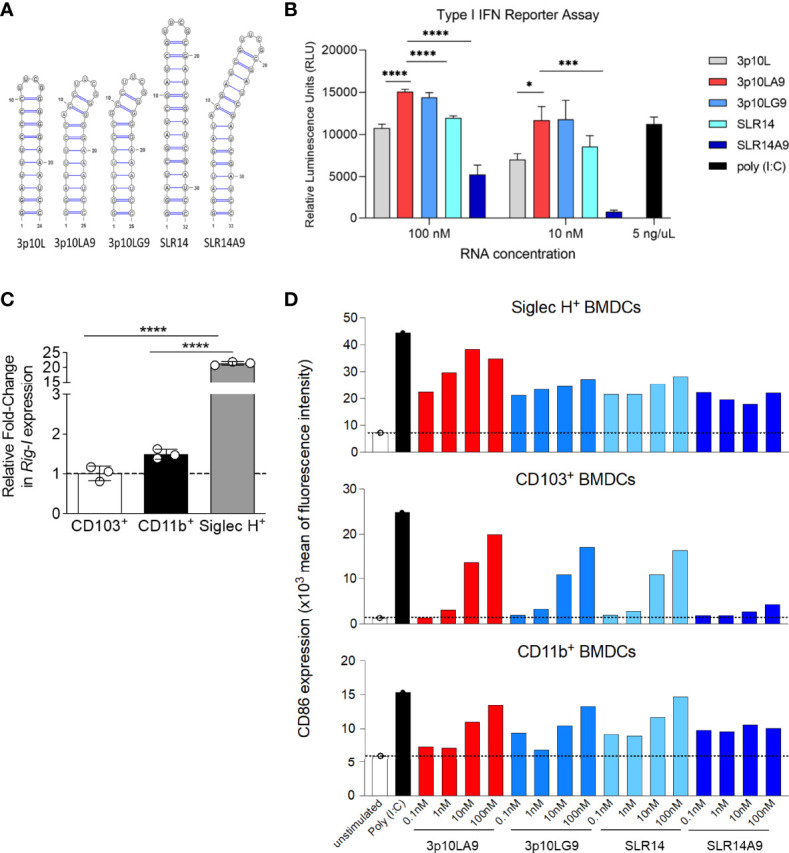
BM-derived DC maturation is induced by dsRNA hairpins **(A)** Sequence and secondary structure of the dsRNA hairpins used in this study. **(B)** Type I IFN related relative luminescence units (RLU) of A549-Dual™ supernatant at 24h post dsRNA hairpin treatment. Poly (I:C) treatment (5 ng/µL) as positive control (n = 3). * P < 0.05; *** P <0.001;**** P < 0.0001; two-tailed t-Student’s Test. These data are representative of three independent experiments. **(C)** Quantitative PCR analysis of RIG-I expression in BM-derived sorted pDCs, CD103^+^ and CD11b^+^ DCs. Data represent the mean ± SEM (n = 3). **(D)** BMDCs were stimulated with 4 different concentrations (0.1 – 100 nM) of dsRNA hairpins (3p10LA9, 3p10LG9, SLR14 and SLR14 A9) and tested 24 h later for CD86 expression by flow cytometry. BM cells were first gated on Siglec H^+^ cells to visualize pDCs, the remaining cells were gated on CD11c^+^MHC II^+^ double positive cells and separated into CD11b and CD103 to delineate two different DC subsets. Unstimulated and Poly I:C (5 μg/ml) stimulated cells were included as negative and positive controls, respectively. Each bar represents one value and the experiment was independently repeated one more time.

Based on previous results, we compared the ability of five dsRNA hairpins to induce a type I IFN response (**Figure 1A**). Three short hairpin candidates (3p10L, 3p10LA9, and 3p10LG9) were from our lab whereas SLR14 was included for comparison (23, 30). We have also tested a fifth RNA, SLR14A9 which has an Adenine insertion at the 9’s position to introduce a bulge similar to 3p10LA9. All RNA candidates were featured with a 5` triphosphorylation and a stem-loop structure varying from 10 to14 bp. All five dsRNA hairpins were transfected to an A549-Dual™ cell line, and the supernatant was taken for luciferase assay after 24h culture. As shown in **Figure 1B**, 3p10LA9 was the most potent candidate in this assay, inducing the highest type I IFN response in a dose ranging from 10 to 100 nM. In addition, SLR14A9 has a reduced ability to trigger IFN induction compared to that of SLR14. Therefore, the A9 insertion mediated enhancement effect is specific to 3p10LA9 but not SLR14A9 in this reporter cell assay.

### Induction of BMDC Maturation by dsRNA Hairpins

We further tested the four dsRNA hairpins (3p10LA9, 3p10LG9, SLR14, and SLR14A9) on BMDCs. First, we confirmed that all BMDCs expressed RIG-I, the receptor for the dsRNA hairpins. pDCs showed higher RIG-I mRNA level compared to CD11b^+^ and CD103^+^ DCs, for which expression levels were much lower (**Figure 1C**). To monitor DC maturation, we stained cells for CD86 expression, a costimulatory molecule that is upregulated during DC maturation. Although all three BMDC subpopulations (CD103^+^ DCs, CD11b^+^ DCs, and pDCs) upregulated CD86 expression in response to all four tested dsRNA hairpins, 3p10LA9 was found to be the most efficient (**Figure 1D**). Therefore, we selected 3p10LA9 as the dsRNA hairpin candidate for all *in vivo* mouse experiments.

### Intranasal dsRNA Hairpin Treatment Augments IFN-α, Interferon-Stimulated Genes and pro-Inflammatory Cytokine/Chemokine Response in the Lower Respiratory Tract

**Figure 2:**
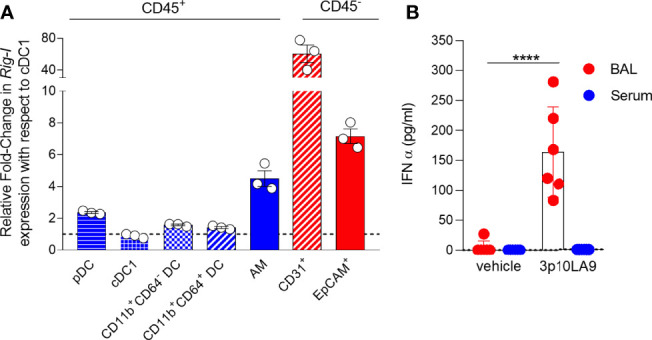
Intranal delivered dsRNA hairpin mediates IFN-α response in the lungs. **(A)** Quantitative PCR (qPCR) analysis of RIG-I expression in sorted lung AM, pDCs, CD103^+^ DCs, CD11b^+^CD64^-^ DCs, and CD11b^+^CD64^+^ DCs as well as EpCAM^+^ epithelial cells and CD31^+^ endothelial cells. **(B)** IFN-α BAL and serum levels were measured by sandwich ELISA 14 h after intranasal delivery (control: vehicle alone; treated group: 3p10LA9). The error bars represent the SD (n= 6 mice).;**** P <0.0001; two-tailed t-Student’s Test.

To test the potential of 3p10LA9 as a candidate adjuvant for intranasal delivery, we first profiled the expression levels of the cytosolic sensor RIG-I in myeloid cells and in epithelial and endothelial cells obtained from lungs of untreated mice. All lung DCs (pDCs, CD103^+^ cDC1, CD11b^+^ cDC2, CD11b^+^CD64^+^ DCs) and alveolar macrophages (AM) expressed RIG-I mRNA, with AM showing the highest levels (**Figure 2A**). Notably, the cytosolic sensor RIG-I was highly expressed in EpCAM^+^ epithelial cells and even more in CD31^+^ endothelial cells, which showed around 7-fold and 60-fold higher expression levels, respectively, compared to pulmonary DCs (**Figure 2A**). Therefore, the RIG-I agonist 3p10LA9 can potentially target both myeloid lung cells and non-hematopoietic cells, such as epithelial and endothelial cells. In fact, when 3p10LA9 was applied intranasally, significantly increased levels of IFN-α were measured in the BAL but not in the serum, suggesting that this type of delivery mainly caused a response locally in the respiratory tract but not systemically (**Figure 2B**).

**Figure 3:**
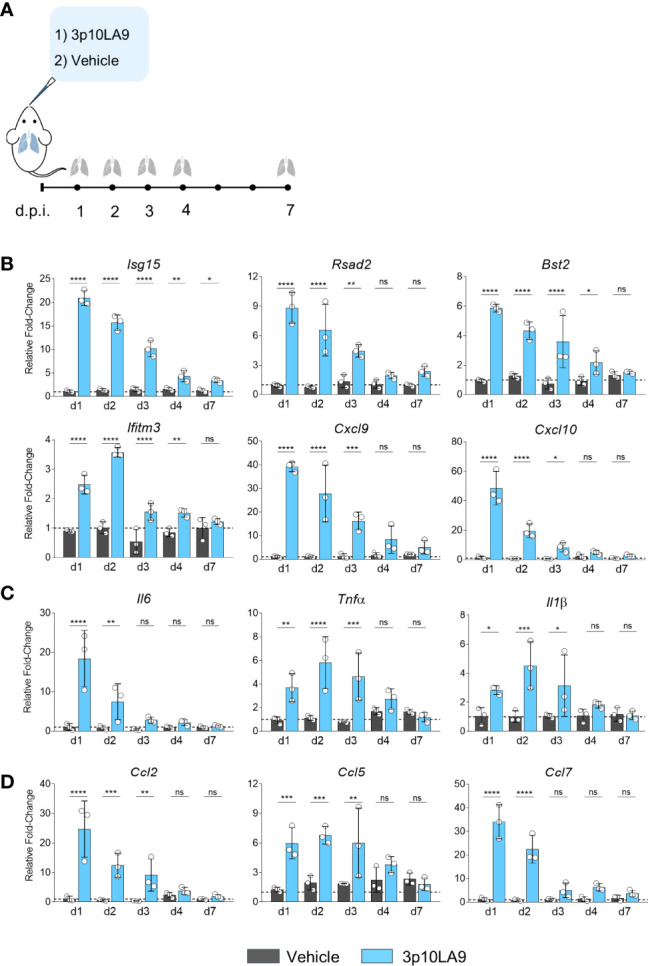
Transient ISG and pro-inflammatory response induced by dsRNA hairpins. **(A)** Intranasal treatment protocol in C57BL/6J mice. **(B-D)** Relative fold change in gene expression of indicated ISGs, cytokines, and chemokines from lung homogenates at d0 (dotted line), d1, d2, d3, d4, and d7 were measured by qRT-PCR obtained from mice treated with 3p10LA9 (treated group, light blue) and vehicle alone (control group, grey). The error bars represent the SEM (n= 3 mice). * P <0.05; *** P <0.001;**** P <0.0001; ns, not significant; two-tailed t-Student’s Test.

To assess 3p10LA9-induced changes in interferon-stimulated genes (ISGs), cytokine- and chemokine expression in the lungs, 8- to 10-week-old C57BL/6J were mice were treated *via* the intranasal route with a single dose of either 3p10LA9 (25 μg/25 μl/mouse) or vehicle alone (**Figure 3A**). At 1, 2, 3, 4 and 7 days post-treatment, mice were euthanized, and lungs were harvested for qPCR analysis of various ISGs, pro-inflammatory cytokines as well as monocyte-attracting chemokines. We found that 3p10LA9 induced a strong transient ISG response in the lower respiratory tract of mice. Expression levels of several ISGs (*Isg15, Rsad2, Bst2, Iftim3, Cxcl9, Cxcl10*) rapidly increased 24 h after 3p10LA9 delivery and declined gradually over the next 3 days, returning to normal levels between 4 to 7 days after the treatment (**Figure 3B**). Moreover, higher pro-inflammatory cytokines mRNA levels were measured in the lung of 3p10LA9-treated animals compared to vehicle-treated control animals. Interleukin *Il6* levels were 20-fold increased, *Tnfα* was increased 4 to 6-fold followed by *Il1β*, which showed a 2-fold increase (**Figure 3C**). In addition, mice treated with 3p10LA9 showed an augmented monocyte-attracting chemokine response within 24 h after intranasal delivery. In particular, *Ccl2* and *Ccl7* mRNA expression was substantially higher in mice receiving intranasal 3p10A9 than in control mice (**Figure 3D**). Taken together, dsRNA hairpin intranasal delivery leads to strong transient ISGs, pro-inflammatory and chemokine responses in the lower respiratory tract.

### Intranasal dsRNA Hairpin Delivery Induces Transient Changes in the Pulmonary Myeloid Cell Composition and Leads to Monocyte Infiltration

**Figure 4:**
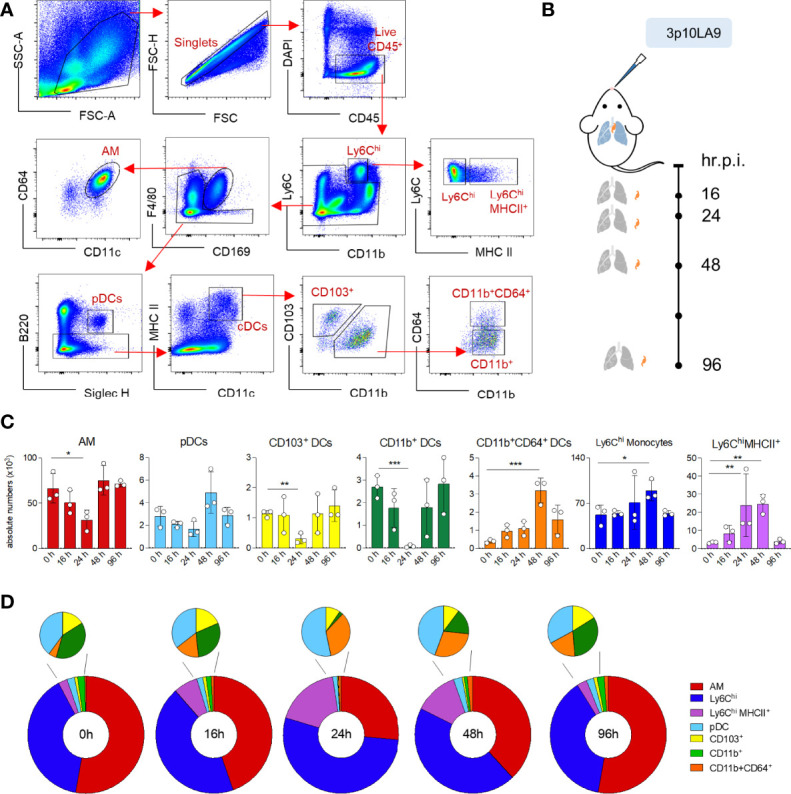
Impact of intranasal dsRNA hairpin treatment on the pulmonary myeloid cell landscape. **(A)** Representative flow cytometry dot plots showing the gating strategy for the myeloid cell landscape in lungs of 8-week-old C57BL/6J mice. AM: F4/80^hi^CD169^+^; monocytes: CD11b^+^Ly6C^high^MHCII^-^; MHCII^+^ monocyte: CD11b^+^Ly6C^high^MHCII^+^; pDCs: B220^+^Siglec-H^+^; cDCs: CD11c^hi^MHCII^+^; CD103^+^ cDC1s: CD103^+^CD11b^-^; CD11b^+^ cDC2s: CD103^-^CD11b^+^CD64^-^; CD11b^+^CD64^+^ cDCs: CD103^-^CD11b^+^CD64^+^. **(B)** Intranasal treatment protocol and time points of lung collection. **(C)** Absolute numbers of pulmonary AM, pDCs, CD103^+^ DCs, CD11b^+^ DCs, CD11b^+^CD64^+^ DCs, Ly6C^hi^ monocytes and Ly6C^hi^MHCII^+^ monocytes at 0h, 16h, 24h, 48h and 96 h post inoculation. The error bars represent the SEM (n= 3 mice). * P <0.05; ** P <0.01; *** P <0.001; two-tailed t-Student’s Test. **(D)** Donut chart showing distribution of the absolute numbers of AM, DC subpopulations and monocytes following 0, 16, 24, 48 and 96 h post intranasal delivery. Values are shown in panel **(C)**.

To investigate the impact of an intranasal 3p10LA9 inoculation on the kinetics of AM, distinct pulmonary monocytes, and DC subpopulations (**Figure 4A**), flow cytometry analysis was performed at different time points (16, 24, 48, and 96 h) post-treatment (**Figure 4B**). In our multiparameter flow cytometry, we included a panel of myeloid markers to delineate AM (F4/80 and CD169), monocytes (Ly6C), activated monocytes (Ly6C and MHC II), conventional DCs (cDCs) (CD11c and MHC II), plasmacytoid DCs (Siglec H and B220), cDC1 (CD103), cDC2 (CD11b) and monocyte-derived DCs (CD64). AM, likely the first cells encountering the RIG-I ligand in the alveolar space showed a significant reduction in numbers 24 h post-treatment, with numbers returning to that of control mice within 48 h (**Figure 4C**, first left panel, **Figure 4D**). Similar changes were observed in pDC numbers, though the slight reduction at 24 h was not significant (**Figure 4C**, second left panel). Both pulmonary CD103^+^ and CD11b^+^ DC numbers were significantly but transiently diminished at 24 h after the intranasal inoculation, returning to baseline one day later, whereas CD11b^+^CD64^+^ DCs, almost absent at steady-state condition, peaked at 48 h post-treatment and were still elevated at 96 h (**Figures 4C, D**). At the same time, the intranasal 3p10LA9 delivery caused a significant infiltration of monocytes. These infiltrating Ly6C^hi^ and Ly6C^hi^MHCII^+^ monocytes peaked at 48 h and declined to steady-state levels within 96 h (**Figure 4C**, right panels, **Figure 4D**). Overall, a single dose of intranasal 3p10LA9 delivery caused a significant reduction of pulmonary CD103^+^ and CD11b^+^ DCs, and a concomitant infiltration of monocyte-related cells into the lungs.

### RIG-I Agonist-Dependent Monocyte Recruitment Leads to a Transient Inflammatory Response in the Lungs

**Figure 5:**
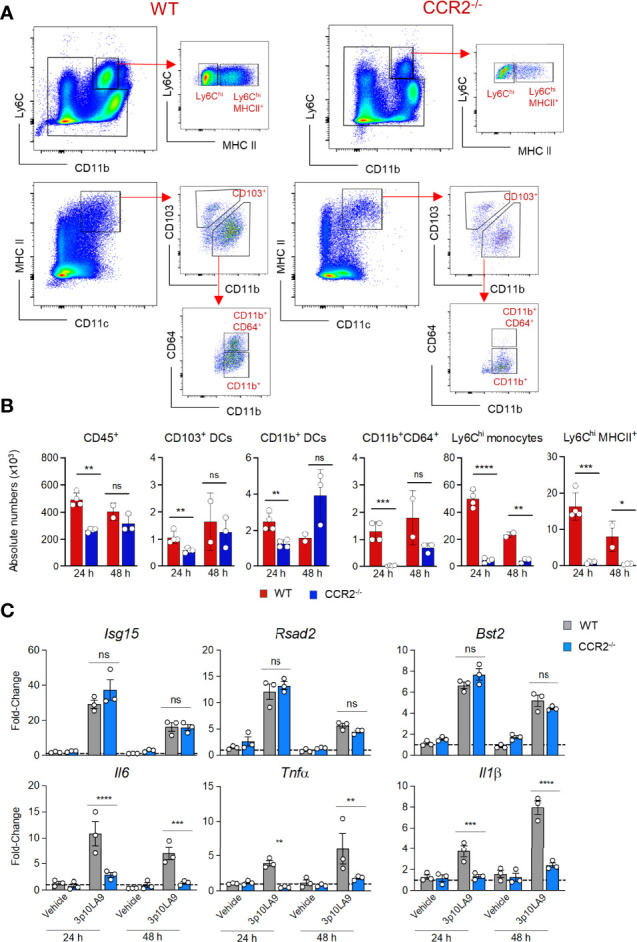
RIG-I agonist-dependent monocyte recruitment leads to a transient inflammatory response in the lungs. **(A)** Representative flow cytometry dot plots showing pulmonary Ly6C^hi^ monocytes and Ly6^hi^HCII^+^ monocytes and distinct DC subpopulations (CD103^+^ DCs, CD11b^+^ DCs, CD11b^+^CD64^+^ DCs) of intranasally 3p10LA9 treated WT (left panel) and CCR2^-/-^ mice (right panel). Lungs were collected 24 h post-delivery. **(B)** Absolute numbers of pulmonary CD45^+^ cells, CD103^+^ DCs, CD11b^+^ DCs, CD11b^+^CD64^+^ DCs, Ly6C^hi^ monocytes and Ly6^hi^MHCII^+^ monocytes in WT and CCR2^-/-^ mice. Lungs were collected 24 h and 48 h post-3p10LA9 treatment. The error bars represent the SEM (n= 2-4 mice). * P < 0.05; ** P < 0.01; *** P < 0.001;**** P < 0.0001; ns, not significant; two-tailed t-Student’s Test. **(C)** Relative fold change in gene expression of indicated ISGs and cytokines was measured by qPCR. Lung homogenates were obtained from WT and CCR2^-/-^ mice 24 h and 48 h post-treatment (3p10LA9 and only vehicle).

We next investigated whether the infiltrating monocytes were the cause for the transient inflammatory response measured in the lower respiratory tract of dsRNA hairpin treated mice. CCR2^-/-^ mice, known for defective monocyte recruitment into the periphery, and WT mice were intranasally treated with 3p10LA9. Both mouse groups were analysed 24 h and 48 h post-treatment by flow cytometry for distinct lung myeloid cell composition, and by qPCR for pulmonary ISG- and inflammatory cytokine expression. As expected in the absence of CCR2, all monocyte-derived cell populations, including Ly6C^hi^monocytes, Ly6C^hi^MHC II^+^ monocytes, and CD11b^+^CD64^+^ DCs, were almost absent in the lungs obtained from CCR2^-/-^ mice (**Figures 5A, B**). At the same time, the pro-inflammatory cytokine response at 24 and 48 h post-treatment was completely abrogated in CCR2^-/-^ mice (**Figure 5C**). *Il6*, *Tnfα* and *Il1β* mRNA levels were significantly reduced in the lungs of 3p10LA9-treated CCR2^-/-^ mice compared to WT controls (**Figure 5C**, lower panel). In contrast, the ISG response was not affected by the absence of monocytes since lungs from both experimental groups showed comparable mRNA levels of *Isg15, Rsad2, and Bst2* (**Figure 5C**, upper panel). Taken together, the pro-inflammatory infiltrating monocytes are the main cause for the transient inflammatory response detected in the lungs of intranasally treated mice.

### Intranasal dsRNA Hairpin Delivery Leads to DC Maturation in the Lungs

**Figure 6:**
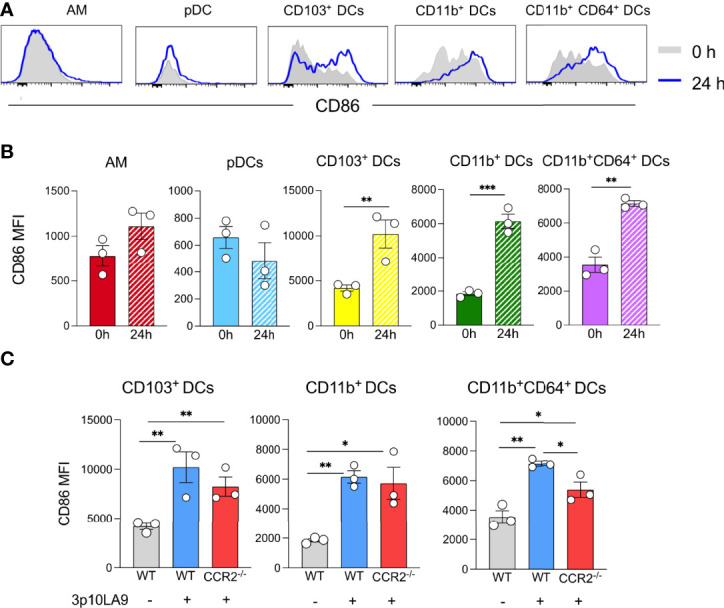
Intranasal dsRNA hairpin delivery induces DC maturation in the lungs. **(A)** Representative flow cytometry histogram showing the expression levels of the costimulatory molecule CD86 on AM, pDCs, CD103^+^ DCs, CD11b^+^ DCs, CD11b^+^CD64^+^ DCs. Mice were intranasally inoculated and lungs collected 24 h later. Grey shaded histogram: untreated; blue line histogram: 3p10LA9. **(B)** Bar chart representing the mean of fluorescence intensity of CD86 on pulmonary AM, pDCs, CD103^+^ DCs, CD11b^+^ DCs, CD11b^+^CD64^+^ DCs from 0h untreated and 24 h treated WT mice. The error bars represent the SEM (n= 3 mice). ** P <0.01; *** P <0.001; two-tailed t-Student’s Test. These data are representative of three independent experiments **(C)** Bar chart representing the mean of fluorescence intensity of CD86 on pulmonary CD103^+^ DCs, CD11b^+^ DCs, and CD11b^+^CD64^+^ DCs from untreated and 24 h treated WT and CCR2^-/-^ mice. Grey bars: WT untreated; blue bars: WT mice, 24 h intranasal 3p10LA9 delivery; red bars: CCR2^-/-^ mice, 24 h intranasal 3p10LA9 delivery; The error bars represent the SEM (n= 3 mice). * P <0.05; ** P <0.01; two-tailed t-Student’s Test.

We next investigated whether intranasal dsRNA hairpin delivery induced maturation of lung AM and pulmonary DC subpopulations 24 h post-treatment. The expression of the costimulatory molecule CD86, as a parameter for DC maturation, was analysed by flow cytometry. Upon 3p10LA9 treatment, AM and pDCs did not show any increase of CD86 expression on their cell surface, whereas CD103^+^, CD11b^+^, and CD11b^+^CD64^+^ DCs upregulated the expression of this costimulatory molecule on their cell surface (**Figures 6A, B**). These results showed that DC subpopulations were undergoing a significant maturation process after intranasal 3p10LA9 delivery. CD86 upregulation was detectable also in DCs isolated from CCR2^-/-^ lungs (**Figure 6C**); therefore, the maturation process was independent of the inflammatory microenvironment in the lungs caused by 3p10LA9.

### DC Traffic to the Lung-Draining Lymph Node Following Intranasal dsRNA Hairpin Treatment

**Figure 7:**
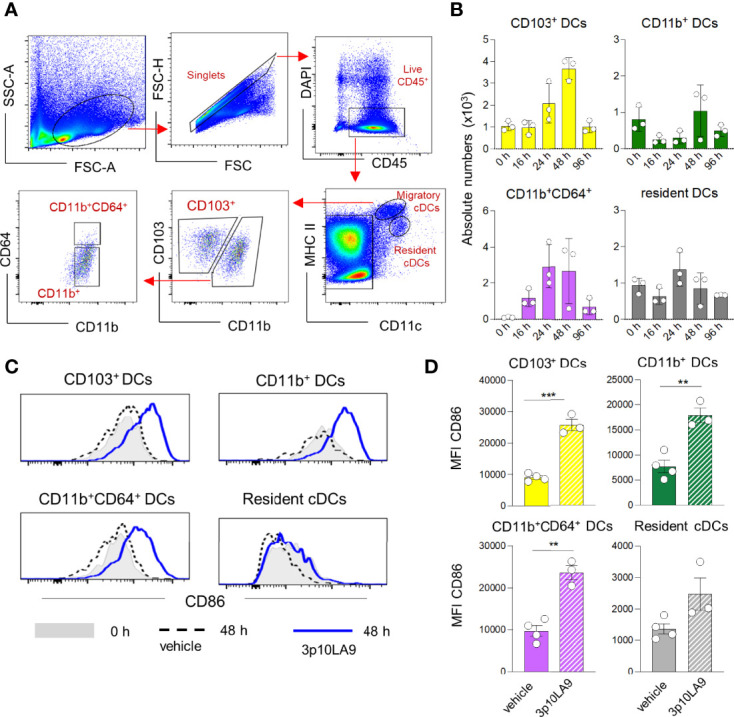
Increased numbers of migratory DC in LN of intranasally treated mice. **(A)** Representative flow cytometry dot plots showing the gating strategy for distinct resident MHCII^+^CD11c^++^ and migratory MHCII^++^CD11c^+^ DC subpopulations (CD103^+^ DCs, CD11b^+^ DCs, CD11b^+^CD64^+^ DCs). **(B)** Intranasal treatment protocol and time points of LN collection as described in **Figure 4B**. Absolute numbers of LN CD103^+^ DCs, CD11b^+^ DCs, CD11b^+^CD64^+^ DCs at 0h, 16h, 24h, 48h and 96 h post intranasal 3p10LA9 inoculation. The error bars represent the SEM (n= 3 mice). Some of the timepoints (0h, 24 and 48 h) have been confirmed by two independent experiments. **(C)** Representative flow cytometry histogram showing the expression levels of the costimulatory molecule CD86 on LN CD103^+^ DCs, CD11b^+^ DCs, CD11b^+^CD64^+^ DCs and resident DCs. Mice were intranasally inoculated and LN collected 48 h later. Grey shaded histogram: untreated; dash line histogram: vehicle alone; blue line histogram: 3p10LA9. **(D)** Bar chart representing the mean of fluorescence intensity of CD86 on LN CD103^+^ DCs, CD11b^+^ DCs, CD11b^+^CD64^+^ DCs and resident DCs from inoculated WT mice (vehicle alone and 3p10LA9). The error bars represent the SEM (n= 3 mice). **P < 0.01; ***P < 0.01; two-tailed t-Student’s Test.

Due to the DC maturation process initiated by the intranasal 3p10LA9 delivery, we next assessed the presence of migratory DCs in lung-draining mediastinal LN. Cells were profiled by flow-cytometry analysis (**Figure 7A**) and absolute numbers were calculated (**Figure 7B**).

Intranasal 3p10LA9 treatment caused an influx of migratory DCs including CD103^+^, CD11b^+^ and CD11b^+^CD64^+^ DCs peaking between 24-48 h (**Figure 7B**). Resident DC numbers were not significantly affected. Cell numbers of migratory cells returned to values seen in naïve mice within 96 h. The maturation status of migratory and resident DCs was compared between control and intranasally 3p10LA9-treated mice. At 48 h post-inoculation, when migratory DC numbers reached the peak in the draining LN, the CD86 expression levels significantly increased on all three migratory DC subpopulations compared to the control counterparts (**Figures 7C, D**). Minimal maturation based on CD86 was observed in LN resident DCs. Taken together, a single dose of intranasal 3p10LA9 promotes a transient accumulation of activated migratory DCs into the lung-draining mediastinal LN within the first 48 h.

### Intranasally Administered dsRNA Hairpins Control Pulmonary Viral Burden in Influenza-Infected Mice

**Figure 8:**
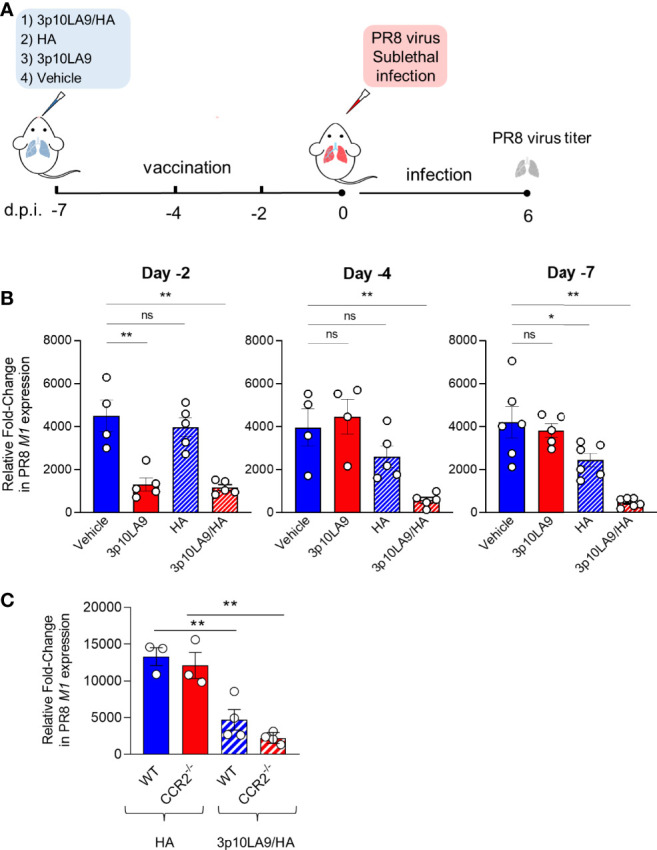
Intranasal 3p10LA9 delivery restrains viral pulmonary load in influenza-infected mice. **(A)** Schematic intranasal 3p10LA9 delivery and IAV sublethal infection protocol. The RIG-I agonist 3p10LA9 was intranasally administered on day 2, day 4 and day 7 before PR8 viral challenge. **(B)** On day 6 post-infection, lung virus load was measured by relative quantification of M1 viral protein in infected mice which have been immunized with (1) vehicle alone (2) 3p10LA9 (3) HA or (4) HA/3p10LA9 at day -2, -4 and -7, respectively. The error bars represent the SEM (n= 4-6 mice/group). * P < 0.05; ** P < 0.01; ns: not significant. One-way ANOVA with Tukey’s multiple comparison test. **(C)** Lung virus load was measured by relative quantification of M1 viral protein in infected WT and CCR2^-/-^ mice which have been immunized with HA mixed with 3p10LA9 (HA/3p10LA9) or HA alone on day 6 post-infection. The error bars represent the SEM (n= 3-4 mice/group). ** P <0.01; One-way ANOVA with Tukey’s multiple comparison test.

To determine the adjuvant efficacy of dsRNA hairpins in an intranasal vaccine formulation, we used 3p10LA9 alone or in combination with influenza A hemaglutinin (HA) as a model antigen and treated mice at three different time points (day -2, -4 and -7) relative to a sublethal PR8 influenza virus challenge (**Figure 8A**). We vaccinated mice *via* the intranasal route with (1) vehicle alone, (2) 3p10LA9, (3) HA or (4) HA/3p10LA9 (**Figure 8A**) and tested their efficacy in reducing the pulmonary virus burden. Lungs were harvested at 6 days post-infection and lung virus titer was assessed by relative quantification of the M1 viral protein *via* qPCR analysis. All infected- unvaccinated control groups showed a 2000-4000 fold change in pulmonary virus loads compared to uninfected WT mice. When mice were infected 2 days post-immunization both 3p10LA9 and HA/3p10LA9 treatments significantly diminished the pulmonary virus load, which indicates that the induced virus reduction is antigen-independent and supported by the strong transient ISG response elicited by the intranasally applied dsRNA hairpins (**Figure 8B**, left). On the contrary, when mice were infected at day 4 and day 7 post-treatment the 3p10LA9 efficacy required the presence of the HA antigen, since the viral burden in the lungs of mice treated with 3p10LA9 and vehicle alone were comparatively high and the pulmonary virus load was only significantly reduced when HA was co-administered to 3p10LA9 (**Figure 8B**, centre and right). Most likely, the viral load reduction was mediated indirectly through the ISG-activated DCs, rather than directly through the dsRNA hairpin-induced short-lived transient ISG response which vanished 3 days after treatment.

To elucidate the contribution of the infiltrating monocytes, WT and CCR2^-/-^ mice were vaccinated both with the HA/3p10LA9 adjuvant combination. As a control group, WT mice were treated with HA. Virus load was measured at 6 days post-PR8 IAV infection. Both vaccinated mouse groups showed lower virus loads than control mice (**Figure 8C**), indicating that the monocytes and their induced pulmonary inflammatory response are not crucial to reduce the viral load.

## Discussion

Adjuvants are sensed primarily by innate immune cells, including DCs. Once activated, these antigen-presenting cells initiate and efficiently support antigen-specific adaptive immune responses required to eradicate invading pathogens. Here we show that synthetic RIG-I agonists, affect the pulmonary myeloid cell landscape and control influenza viral load after sublethal IAV infection. In previous studies, mainly intravenous or intramuscular but rarely intranasal routes (1, 17, 25, 31) were used to deliver RIG-I agonists. In our study, we opted for the intranasal mucosal delivery approach instead of the widely used systemic delivery. This strategy has many advantages over conventional parenteral treatment because intranasal delivery generates strong local immune responses at mucosal sites of viral exposure, therefore the best approach to monitor myeloid cell perturbations within the respiratory tissue.

To investigate the anti-viral and adjuvant properties of dsRNA hairpins in intranasal vaccination, we have focused on one particular dsRNA hairpin, called 3p10LA9, which showed to be, among the tested dsRNA hairpins, the best candidate in stimulating a type I IFN response both in the type I IFN reporter cell line and in primary murine BM-derived DCs.

In the respiratory tract, RIG-I is expressed by various myeloid cells, including different DC subpopulations and alveolar macrophages, the latter located in the alveolar space and therefore constituting the first line of defence against invading pathogens. Similar to other PRRs, such as TLRs, we show that RIG-I expression is not limited to immune cells. Non-immune lung epithelial and endothelial cells expressed the highest levels of RIG-I. This is in alignment with another study showing that i.v injected fluorochrome labelled SLR14 dsRNA hairpin was taken up by both immune and non-immune cells, with epithelial cells and macrophages showing the strongest uptake (17). Thus, a diverse spectrum of cells of the respiratory tract can sense RIG-I agonists and contribute to the strong ISG response observed upon intranasal treatment with dsRNA hairpins.

These 3p10LA9-induced changes in the lung microenvironment, including enhanced levels of monocyte-attracting chemokines and pro-inflammatory cytokines, promoted not only a significant influx of monocytes into the respiratory tract of 3p10LA9-treated mice but also a transient reduction of alveolar macrophages and pulmonary DCs. The depletion of alveolar macrophages within the first 24 h could be driven by inflammatory cell death, a phenomenon commonly known as macrophage disappearance reaction observed in other microbial infections (32, 33). Macrophage cell death can precede and initiate the recruitment of monocytes (34). In addition, 3p10LA9 treatment promotes DC maturation in the lungs and enhances their migration from the lungs into the draining LN within the first two days post-delivery.

The infiltrating monocytes supported this transient local pulmonary inflammation since pro-inflammatory cytokines, such as IL-6, TNF-α and IL-1β, could only be detected in WT mice but not in CCR2^-/-^ mice which are known for their impaired inflammatory-mediated monocyte recruitment. The infiltration of monocytes into the lower respiratory tract upon intranasal treatment with 3p10LA9 was transient, and it lasted only a few days without inducing prolonged local inflammation, which is an important aspect of safe vaccines.

Under normal homeostasis, BM-derived circulating monocytes not only replenish and maintain macrophages in different tissues and organs (35), but also support a migratory cross-presenting CD64^+^ DC subpopulation which we have previously identified in several tissues including the respiratory tract (36). Infiltrating monocytes during infection and inflammation can give rise to monocyte-derived DCs, with an apparent inflammatory cDC2 phenotype (37). Similarly, our data show that intranasal delivery of 3p10LA9 led to a significant increase of LyC6^hi^ and Ly6C^hi^MHCII^+^ activated monocytes, but also CD64^+^ DCs in the lungs and the draining LNs. Based on our results, these cells are dispensable and not crucial for virus control after challenge since vaccinated CCR2^-/-^ mice, lacking these monocyte-dependent infiltrating cells after intranasal 3p10LA9 delivery, still showed reduced virus load upon IAV infection. Thus, the strong ISG response obtained in the respiratory tract microenvironment upon dsRNA hairpin delivery is the main driver for DC maturation and migration to the LNs. Notably, IL-6 and TNF-α were not required for virus load reduction in our experimental setup. This conclusion is supported by the fact that CCR2^-/-^ mice lacking pulmonary pro-inflammatory response showed a reduced viral load upon intranasal dsRNA hairpin delivery compared to WT mice. Our data suggest a central contribution of migratory CD103^+^ and CD11b^+^ DCs in promoting 3p10LA9-induced IAV load reduction and show that LN resident DCs do not seem to play a direct role since their maturation status is not or minimally affected by the intranasal adjuvant delivery.

Interestingly, mice deficient in RIG-I-MAVS pathways display defects in migratory DCs, their viral antigen presentation, and CD8^+^ and CD4^+^ T cell activation during IAV infection, which leads to delayed clearance of the IAV from the lungs of RIG-I^-/-^ mice (38). During IAV infection, the maturation of CD103^+^ DCs, the main cross-presenting DC subpopulation, is affected in RIG-I^-/-^ mice, whereas CD11b^+^ DCs seem to be less affected in these mice. The observed lack of virus control correlates with a reduced Type I IFN, underlying the importance of this signalling pathway in mediating innate and adaptive anti-viral responses not only during “natural” viral infections but also during effective anti-viral vaccination strategies. Similar to our results, intranasal delivery of chemically modified bent RNA duplexes in combination with inactivated PR8 virus protected mice infected with a lethal dose of PR8 virus (39). The vaccinated mice showed higher anti-IAV IgG titers than mice treated without the adjuvant, demonstrating that RIG-I agonists enhance adaptive anti-viral responses (39). Moreover, incorporating RIG-I agonist 5’pppRNA into influenza virus-like particles expressing H5N1 HA and neuraminidase (NA) enhanced both cellular and humoral mediated protective responses against H5N1 influenza virus challenge (18). Furthermore, another RIG-I ligand, double-stranded 3pRNA, was shown to strongly enhance protective CTL-cross-priming against adenoviral infection (40).

Importantly, we have also observed HA-independent short-term reduction in viral load when the RIG-I agonist was applied intranasally shortly before influenza infection. Due to its robust transient ISG response within the first three days, one intranasal administration of 3p10LA9 alone without HA significantly reduced the viral burden in the lungs of influenza-infected mice. Consistent with our data, recent publications demonstrated similar efficacy in prophylactic short-term antiviral immunity not only against influenza (25) but also against SARS-CoV-2 (17, 41). Intravenously injected RIG-I agonists, when injected within 24 h before SARS-CoV-2 challenge, prevented viral infection of the lower respiratory tract in a type I IFN dependent-manner and the protection was independent of adaptive immune responses (17). Although two different administration approaches (intranasal versus i.v.) were used in our and these recent studies, both delivery routes led to the control of the pulmonary viral burden when applied shortly before the viral challenge.

There are some limitations to this study. Since the focus was to elucidate detailed effects of the RIG-I agonist 3p10LA9 on the early myeloid cell response, including the relevance of the inflammatory response, the adaptive response was not studied for this report. It remains to be established to which extent intranasal RIG-I agonist induces locally secreted IgA in the upper respiratory tract. Further studies will also need to address other potential IFN-I-independent factors inducing short-term protection, and how long the antigen-dependent adjuvant effect of RIG-I protects against re-infection.

In summary, our study suggests that RIG-I agonists, such as dsRNA hairpins, are promising anti-viral prophylactic adjuvants suitable for intranasal delivery. Given the efficient and local response in the lung observed in our study, dsRNA hairpin-based vaccines could provide protection not only against IAV infection but possibly against a broader spectrum of respiratory viral pathogens.

## Data Availability Statement

The datasets presented in this study can be found in online repositories. The names of the repository/repositories and accession number(s) can be found below: The original flow cytometry data have been deposited in the NTU Open Access Data Repository (DR-NTU). https://doi.org/10.21979/N9/XYX2FS.

## Ethics Statement

All studies involving mice were carried out in strict accordance with the recommendations of the National Advisory Committee for Laboratory Animal Research and all protocols were approved by the Institutional Animal Care and Use Committee of the Nanyang Technological University (ARF-SBS/NIE A19050 and A-0375).

## Author Contributions

CR conceived the study and designed the experiments. SN, YW, and TMN performed the experiments. CR, KF, and DL directed and interpreted the experiments. CR, SN, and YW were involved in data analysis. CR wrote the manuscript, and all authors contributed to editing the document. KF and DL secured the funding for the study. All authors contributed to the article and approved the submitted version.

## Funding

This work was supported by a National Medical Research Council (NMRC) grant OFIRG17nov084 awarded to DL.

## Conflict of Interest

DL and KF are inventors on a patent for immRNA applications.

The remaining authors declare that the research was conducted in the absence of any commercial or financial relationships that could be construed as a potential conflict of interest.

## Publisher’s Note

All claims expressed in this article are solely those of the authors and do not necessarily represent those of their affiliated organizations, or those of the publisher, the editors and the reviewers. Any product that may be evaluated in this article, or claim that may be made by its manufacturer, is not guaranteed or endorsed by the publisher.

## References

[1] Wong PT Goff PH Sun RJ Ruge MJ Ermler ME Sebring A. Combined Intranasal Nanoemulsion and RIG-I Activating RNA Adjuvants Enhance Mucosal, Humoral, and Cellular Immunity to Influenza Virus. Mol Pharm (2021) 18(2):679–98. doi: 10.1021/acs.molpharmaceut.0c00315 32491861

[2] Holmgren J Czerkinsky C. Mucosal Immunity and Vaccines. Nat Med (2005) 11(4 Suppl):S45–53. doi: 10.1038/nm1213 15812489

[3] Lavelle EC Ward RW. Mucosal Vaccines - Fortifying the Frontiers. Nat Rev Immunol (2022) 22(4):236–50. doi: 10.1038/s41577-021-00583-2 PMC8312369 34312520

[4] Kato H Sato S Yoneyama M Yamamoto M Uematsu S Matsui K. Cell Type-Specific Involvement of RIG-I in Antiviral Response. Immunity (2005) 23(1):19–28. doi: 10.1016/j.immuni.2005.04.010 16039576

[5] Medzhitov R. Toll-Like Receptors and Innate Immunity. Nat Rev Immunol (2001) 1(2):135–45. doi: 10.1038/35100529 11905821

[6] Hornung V Ellegast J Kim S Brzozka K Jung A Kato H. 5'-Triphosphate RNA is the Ligand for RIG-I. Science (2006) 314(5801):994–7. doi: 10.1126/science.1132505 17038590

[7] Loo YM Gale M Jr. Immune Signaling by RIG-I-Like Receptors. Immunity (2011) 34(5):680–92. doi: 10.1016/j.immuni.2011.05.003 PMC3177755 21616437

[8] Yoneyama M Kikuchi M Natsukawa T Shinobu N Imaizumi T Miyagishi M. The RNA Helicase RIG-I has an Essential Function in Double-Stranded RNA-Induced Innate Antiviral Responses. Nat Immunol (2004) 5(7):730–7. doi: 10.1038/ni1087 15208624

[9] Weber M Gawanbacht A Habjan M Rang A Borner C Schmidt AM. Incoming RNA Virus Nucleocapsids Containing a 5'-Triphosphorylated Genome Activate RIG-I and Antiviral Signaling. Cell Host Microbe (2013) 13(3):336–46. doi: 10.1016/j.chom.2013.01.012 PMC5515363 23498958

[10] Pichlmair A Schulz O Tan CP Naslund TI Liljestrom P Weber F. RIG-I-Mediated Antiviral Responses to Single-Stranded RNA Bearing 5'-Phosphates. Science (2006) 314(5801):997–1001. doi: 10.1126/science.1132998 17038589

[11] Platanias LC. Mechanisms of Type-I- and Type-II-Interferon-Mediated Signalling. Nat Rev Immunol (2005) 5(5):375–86. doi: 10.1038/nri1604 15864272

[12] Stark GR Darnell JE Jr. The JAK-STAT Pathway at Twenty. Immunity (2012) 36(4):503–14. doi: 10.1016/j.immuni.2012.03.013 PMC3909993 22520844

[13] Rehwinkel J Gack MU. RIG-I-Like Receptors: Their Regulation and Roles in RNA Sensing. Nat Rev Immunol (2020) 20(9):537–51. doi: 10.1038/s41577-020-0288-3 PMC7094958 32203325

[14] Pulendran B Arunachalam PS O'Hagan DT. Emerging Concepts in the Science of Vaccine Adjuvants. Nat Rev Drug Discovery (2021) 20(6):454–75. doi: 10.1038/s41573-021-00163-y PMC8023785 33824489

[15] Jiang X Muthusamy V Fedorova O Kong Y Kim DJ Bosenberg M. Intratumoral Delivery of RIG-I Agonist SLR14 Induces Robust Antitumor Responses. J Exp Med (2019) 216(12):2854–68. doi: 10.1084/jem.20190801 PMC6888973 31601678

[16] Ziegler A Soldner C Lienenklaus S Spanier J Trittel S Riese P. A New RNA-Based Adjuvant Enhances Virus-Specific Vaccine Responses by Locally Triggering TLR- and RLH-Dependent Effects. J Immunol (2017) 198(4):1595–605. doi: 10.4049/jimmunol.1601129 28077601

[17] Mao T Israelow B Lucas C Vogels CBF Gomez-Calvo ML Fedorova O. A Stem-Loop RNA RIG-I Agonist Protects Against Acute and Chronic SARS-CoV-2 Infection in Mice. J Exp Med (2022) 219(1):e20211818. doi: 10.1084/jem.20211818 34757384 PMC8590200

[18] Beljanski V Chiang C Kirchenbaum GA Olagnier D Bloom CE Wong T. Enhanced Influenza Virus-Like Particle Vaccination With a Structurally Optimized RIG-I Agonist as Adjuvant. J Virol (2015) 89(20):10612–24. doi: 10.1128/JVI.01526-15 PMC4580177 26269188

[19] Heidegger S Kreppel D Bscheider M Stritzke F Nedelko T Wintges A. RIG-I Activating Immunostimulatory RNA Boosts the Efficacy of Anticancer Vaccines and Synergizes With Immune Checkpoint Blockade. EBioMedicine (2019) 41:146–55. doi: 10.1016/j.ebiom.2019.02.056 PMC6444128 30852164

[20] Doener F Hong HS Meyer I Tadjalli-Mehr K Daehling A Heidenreich R. RNA-Based Adjuvant CV8102 Enhances the Immunogenicity of a Licensed Rabies Vaccine in a First-in-Human Trial. Vaccine (2019) 37(13):1819–26. doi: 10.1016/j.vaccine.2019.02.024 30797640

[21] Olagnier D Scholte FE Chiang C Albulescu IC Nichols C He Z. Inhibition of Dengue and Chikungunya Virus Infections by RIG-I-Mediated Type I Interferon-Independent Stimulation of the Innate Antiviral Response. J Virol (2014) 88(8):4180–94. doi: 10.1128/JVI.03114-13 PMC3993760 24478443

[22] Ho V Yong HY Chevrier M Narang V Lum J Toh YX. RIG-I Activation by a Designer Short RNA Ligand Protects Human Immune Cells Against Dengue Virus Infection Without Causing Cytotoxicity. J Virol (2019) 93(14):e00102-19. doi: 10.1128/JVI.00102-19 31043531 PMC6600207

[23] Yong HY Zheng J Ho VCY Nguyen MT Fink K Griffin PR. Structure-Guided Design of Immunomodulatory RNAs Specifically Targeting the Cytoplasmic Viral RNA Sensor RIG-I. FEBS Lett (2019) 593(21):3003–14. doi: 10.1002/1873-3468.13564 31369683

[24] Luke JM Simon GG Soderholm J Errett JS August JT Gale M Jr.. Coexpressed RIG-I Agonist Enhances Humoral Immune Response to Influenza Virus DNA Vaccine. J Virol (2011) 85(3):1370–83. doi: 10.1128/JVI.01250-10 PMC3020507 21106745

[25] Coch C Stumpel JP Lilien-Waldau V Wohlleber D Kummerer BM Bekeredjian-Ding I. RIG-I Activation Protects and Rescues From Lethal Influenza Virus Infection and Bacterial Superinfection. Mol Ther (2017) 25(9):2093–103. doi: 10.1016/j.ymthe.2017.07.003 PMC5589155 28760668

[26] Goulet ML Olagnier D Xu Z Paz S Belgnaoui SM Lafferty EI. Systems Analysis of a RIG-I Agonist Inducing Broad Spectrum Inhibition of Virus Infectivity. PLoS Pathog (2013) 9(4):e1003298. doi: 10.1371/journal.ppat.1003298 23633948 PMC3635991

[27] Yong HY Luo D. RIG-I-Like Receptors as Novel Targets for Pan-Antivirals and Vaccine Adjuvants Against Emerging and Re-Emerging Viral Infections. Front Immunol (2018) 9:1379. doi: 10.3389/fimmu.2018.01379 29973930 PMC6019452

[28] Purnama C Ng SL Tetlak P Setiagani YA Kandasamy M Baalasubramanian S. Transient Ablation of Alveolar Macrophages Leads to Massive Pathology of Influenza Infection Without Affecting Cellular Adaptive Immunity. Eur J Immunol (2014) 44(7):2003–12. doi: 10.1002/eji.201344359 24687623

[29] Ng SL Teo YJ Setiagani YA Karjalainen K Ruedl C. Type 1 Conventional CD103(+) Dendritic Cells Control Effector CD8(+) T Cell Migration, Survival, and Memory Responses During Influenza Infection. Front Immunol (2018) 9:3043. doi: 10.3389/fimmu.2018.03043 30622538 PMC6308161

[30] Linehan MM Dickey TH Molinari ES Fitzgerald ME Potapova O Iwasaki A. A Minimal RNA Ligand for Potent RIG-I Activation in Living Mice. Sci Adv (2018) 4(2):e1701854. doi: 10.1126/sciadv.1701854 29492454 PMC5821489

[31] Martinez-Gil L Goff PH Hai R Garcia-Sastre A Shaw ML Palese P. A Sendai Virus-Derived RNA Agonist of RIG-I as a Virus Vaccine Adjuvant. J Virol (2013) 87(3):1290–300. doi: 10.1128/JVI.02338-12 PMC3554167 23175362

[32] Barth MW Hendrzak JA Melnicoff MJ Morahan PS. Review of the Macrophage Disappearance Reaction. J Leukoc Biol (1995) 57(3):361–7. doi: 10.1002/jlb.57.3.361 7884305

[33] Ginhoux F Bleriot C Lecuit M. Dying for a Cause: Regulated Necrosis of Tissue-Resident Macrophages Upon Infection. Trends Immunol (2017) 38(10):693–5. doi: 10.1016/j.it.2017.05.009 28642066

[34] Guilliams M Scott CL. Does Niche Competition Determine the Origin of Tissue-Resident Macrophages? Nat Rev Immunol (2017) 17:451–60. doi: 10.1038/nri.2017.42 28461703

[35] Sheng J Ruedl C Karjalainen K. Most Tissue-Resident Macrophages Except Microglia Are Derived From Fetal Hematopoietic Stem Cells. Immunity (2015) 43(2):382–93. doi: 10.1016/j.immuni.2015.07.016 26287683

[36] Sheng J Chen Q Soncin I Ng SL Karjalainen K Ruedl C. A Discrete Subset of Monocyte-Derived Cells Among Typical Conventional Type 2 Dendritic Cells Can Efficiently Cross-Present. Cell Rep (2017) 21(5):1203–14. doi: 10.1016/j.celrep.2017.10.024 29091760

[37] Bosteels C Neyt K Vanheerswynghels M van Helden MJ Sichien D Debeuf N. Inflammatory Type 2 cDCs Acquire Features of Cdc1s and Macrophages to Orchestrate Immunity to Respiratory Virus Infection. Immunity (2020) 52(6):1039–56.e9. doi: 10.1016/j.immuni.2020.04.005 32392463 PMC7207120

[38] Kandasamy M Suryawanshi A Tundup S Perez JT Schmolke M Manicassamy S. RIG-I Signaling Is Critical for Efficient Polyfunctional T Cell Responses During Influenza Virus Infection. PLoS Pathog (2016) 12(7):e1005754. doi: 10.1371/journal.ppat.1005754 27438481 PMC4954706

[39] Lee MK Kim HE Park EB Lee J Kim KH Lim K. Structural Features of Influenza A Virus Panhandle RNA Enabling the Activation of RIG-I Independently of 5'-Triphosphate. Nucleic Acids Res (2016) 44(17):8407–16. doi: 10.1093/nar/gkw525 PMC5041458 27288441

[40] Hochheiser K Klein M Gottschalk C Hoss F Scheu S Coch C. Cutting Edge: The RIG-I Ligand 3prna Potently Improves CTL Cross-Priming and Facilitates Antiviral Vaccination. J Immunol (2016) 196(6):2439–43. doi: 10.4049/jimmunol.1501958 26819202

[41] Marx S Kummerer BM Grutzner C Kato H Schlee M Renn M. RIG-I-Induced Innate Antiviral Immunity Protects Mice From Lethal SARS-CoV-2 Infection. Mol Ther Nucleic Acids (2022) 27:1225–34. doi: 10.1016/j.omtn.2022.02.008 PMC8841011 35186439

